# Prognostic value of neutrophil to lymphocyte ratio in patients with esophagus cancer receiving neoadjuvant therapy: a systematic review and meta-analysis

**DOI:** 10.3389/fimmu.2025.1615962

**Published:** 2025-10-06

**Authors:** Longwei Ma, Jiaxing He, Ping Li, Long Ma, He Wang, Yanchao Deng

**Affiliations:** ^1^ Department of Thoracic Surgery, The First Affiliated Hospital of Xinjiang Medical University, Urumqi, Xinjiang Uygur Autonomous Region, China; ^2^ Breast and Thyroid Surgery Department, Xinjiang Medical University Affiliated Tumor Hospital, Urumqi, Xinjiang Uygur Autonomous Region, China; ^3^ Department of Otolaryngology Head and Neck Surgery, Yili Friendship Hospital, Yili, Xinjiang Uygur Autonomous Region, China; ^4^ Clinical Medicine Department, Xinjiang Medical University, Urumqi, Xinjiang Uygur Autonomous Region, China

**Keywords:** NLR, EC, NCRT, NCT, os, RFS, PCR

## Abstract

**Background:**

Growing research reveals a relation of the neutrophil-to-lymphocyte ratio (NLR) to clinical outcomes of the esophageal cancer (EC) population undergoing neoadjuvant therapy. However, current findings remain inconclusive and somewhat controversial.

**Methods:**

PubMed, Embase, Web of Science, and the Cochrane Library were thoroughly retrieved until April 22, 2025 to collect studies on the link of NLR to prognosis among the EC population following neoadjuvant therapy. Eligible studies were selected as per predefined eligibility criteria. The primary outcomes encompassed overall survival (OS), recurrence-free survival (RFS), and pathological complete response (pCR). Hazard ratios (HRs) and corresponding 95% confidence intervals (CIs) were pooled for prognostic significance assessment along with subgroup analyses. The evidence was graded via the GRADE method.

**Results:**

11 cohort studies involving 2,220 patients were included in the analysis. The results demonstrated a notable link of risen NLR to less favorable OS (HR = 1.99, 95% CI: 1.43–2.76, P < 0.0001; I² = 88%), shorter RFS (HR = 2.69, 95% CI: 1.77–4.08, P < 0.00001; I² = 47%), and lower pCR rates (OR = 0.67, 95% CI: 0.47–0.94, P = 0.02; I² = 62%). Subgroup analyses by sample size, follow-up length, age, treatment modality, and NLR cut-off value consistently demonstrated a strong correlation between elevated NLR and shortened RFS across all subgroups. Notably, in patients receiving neoadjuvant chemoradiotherapy (NCRT), the link of increased NLR to OS and RFS appeared more robust compared to those receiving neoadjuvant chemotherapy (NCT) alone.

**Conclusion:**

In patients with EC undergoing neoadjuvant therapy, a higher pre-treatment NLR is significantly linked to worse OS and RFS, as well as a lower likelihood of achieving pCR. Therefore, NLR can be a valuable prognostic biomarker in this patient population, potentially aiding clinicians in risk stratification and treatment decision-making.

**Systematic review registration:**

https://www.crd.york.ac.uk/prospero/, identifier CRD42024610088.

## 1 Introduction

Esophageal cancer (EC), one of the most lethal malignancies worldwide, represents the seventh predominant cause of global mortality related to cancer (1). Though there have been notable advancements in therapeutic approaches and critical care in recent decades, the EC population demonstrates one of the least favorable five-year survival outcomes in contrast to other cancers (2). The GLOBOCAN 2020 global cancer statistics showed approximately 604,000 new and 544,000 dead cases attributable to EC, ranking seventh and sixth in incidence and mortality, among all malignancies (3). Notably, nearly half of all new cases and deaths occurred in East Asia (4). Its treatment presents unique challenges owing to the anatomical proximity of the esophagus to the airway and main blood vessels, no serosal layer, and its rich surrounding lymphatic network. Furthermore, patients are commonly diagnosed at a late stage and frequently malnourished, further complicating treatment (5). We accurately determined the stage of esophageal cancer according to the international esophageal cancer diagnostic guidelines, which is important for us to choose the appropriate treatment, so that we can provide individualized treatment pathways for patients with different stages of esophageal cancer (6).In the early stage, surgical resection is still the mainstay and most effective approach. However, due to the insidious onset and aggressive nature of EC, many cases are detected at advanced stages, thereby missing the best opportunity for curative surgery (7). With a better understanding of the disease, various treatment strategies have been developed for the locally advanced EC population, like neoadjuvant chemoradiotherapy (NCRT), neoadjuvant chemotherapy (NCT), as well as immunotherapy with surgery (8). Evidence suggests that NCRT significantly improves overall survival (OS) in locally advanced EC, with clinical benefits observed across different histological subtypes (9); Similarly, several studies have reported a survival advantage with NCT in this patient population (10). In terms of recurrence-free survival (RFS), Zhang et al. demonstrated that neoadjuvant treatment - whether NCRT or NCT - contributes positively to improved outcomes (11); Furthermore, Lewis et al. found that neoadjuvant therapy increases the pathological complete response (pCR) rate compared to surgery alone (12). Hence, early and accurate prognostic assessment is critical for guiding individualized treatment decisions in clinical practice.

Tumor progression is influenced by both tumor-specific factors and the host immune response (13, 14). Therefore, systemic inflammatory markers are prospective prognostic indicators. A low neutrophil-to-lymphocyte ratio (NLR) influences favorable outcomes in various malignancies, including non-small cell lung cancer, gastric cancer, colorectal cancer, and hepatocellular carcinoma (15, 16). Systemic inflammation can be assessed via alterations in peripheral blood cell counts of lymphocytes, monocytes, neutrophils, and platelets. Composite hematological indices, like the NLR and platelet-to-lymphocyte ratio (PLR), have been proposed as accessible prognostication biomarkers. In 2021, Li et al.’s study, which included 127 patients, NLR was a dependable indicator of prognosis for EC sufferers undergoing neoadjuvant therapy (17). Conversely, a contemporaneous study by Anand et al. reported limited predictive value of NLR in this context (18). These conflicting findings underscore the need for further investigation into the prognosis utility of NLR among the EC population after neoadjuvant therapy.

To date, a substantial number of both retrospective and prospective investigations have examined the predictive value of NLR in this context, yet a comprehensive meta-analysis to consolidate the available research findings remains lacking. Therefore, our study unveiled the prognosis relevance of NLR among EC individuals taking neoadjuvant therapy via a systematic review and meta-analysis. GRADE methodology and subgroup analyses were employed to assess the evidence quality and identify possible sources of heterogeneity. Ultimately, this study seeks to present evidence-based recommendations for the clinical adoption of NLR as a readily available hematological biomarker to facilitate prognostic stratification and guide therapeutic decision-making, ameliorating outcomes for the EC population.

## 2 Materials and methods

### 2.1 Literature search

Our study followed the Preferred Reporting Items for Systematic Reviews and Meta-Analyses (PRISMA 2020) guidelines (19). Our study protocol was prospectively registered in the International Prospective Register of Systematic Reviews (PROSPERO; Registration No.: CRD42024610088). LWM and JXH developed the search strategy and independently have searched terms and keywords comprehensively across PubMed, Embase, Web of Science, and the Cochrane Library up to April 22, 2025. A broad range of search terms was employed, such as “EC,” “neoadjuvant therapy,” “neutrophils,” “lymphocytes,” “NCRT,” “NCT,” and “NLR.” The strategy is provided in **Supplementary Table 1**.

### 2.2 Study selection

The inclusion criteria were: (1) EC diagnosis based on endoscopic evaluation and histopathological biopsy; (2) patients received preoperative neoadjuvant therapy, including NCRT or NCT, with commonly used regimens comprising epirubicin and cisplatin with fluorouracil or capecitabine, or epirubicin and oxaliplatin with fluorouracil or capecitabine; (3) evaluating the prognostic utility of NLR on OS, RFS, and pCR; (4) hazard ratio (HR) data with corresponding 95% confidence intervals (CIs) were offered or could be computed; (5) patients were divided into high- and low-NLR cohorts based on a defined cutoff value; (6) only fully published studies were considered. The exclusion criteria were: (1) reviews, commentaries, conference abstracts, and case reports, as well as letters; (2) insufficient information for HRs and 95% CIs computation; (3) no survival outcome data; and (4) duplicate or overlapping data.

Two reviewers (LWM and JXH) independently checked the titles and abstracts, accessed the full texts, and assessed eligibility. Any dissents in the selection process were addressed via discussion and consensus. 34studies published in English were identified through the search strategy applied across the four databases. These studies were initially translated utilizing the professional translation software “ZhiYun,” followed by independent data extraction conducted by two English-proficient investigators (LWM and JXH). Any terminological inconsistencies encountered during translation were resolved in consultation with a third English-language expert (Dr. YCD) to reach a consensus. Data extraction strictly adhered to the PICOS framework, and key prognostic indicators were systematically recorded utilizing standardized data collection forms.

### 2.3 Data extraction

LWM and JXH independently gathered information. Dissents were addressed after consensus. Gathered data comprised the first author, publication year, country (study location), design, sample size, age, duration, pathological stage, treatment, timing of NLR assessment, cutoff value, follow-up length, as well as HRs with 95% CIs for OS, RFS, and pCR.

### 2.4 Quality assessment

The study quality was rated via the Newcastle-Ottawa Scale (NOS) assessing selection, comparability, as well as outcome. The maximum score for each study was 9 points (20). 7–9 denoted high quality (21). Agreement statistics between two authors (LWM and JXH) regarding study selection were performed using Cohen’s kappa statistics and associated 95% CI. Magnitude of agreement was interpreted following guidelines reported by Landis and Koch: slight (0.00-0.20), fair (0.21-0.40), moderate (0.41-0.60), substantial (0.61-0.80), and almost perfect agreement (0.81-1.00) (22).

### 2.5 Statistical analysis

Pooled HRs with corresponding 95% CIs were derived for assessing NLR’s prognosis utility among the EC population who received neoadjuvant therapy. Heterogeneity was examined utilizing Cochran’s Q test and Higgins’ I² statistic (23). Each statistical analysis adopted a random-effects model. To examine the stability of our OS、RFS and pCR findings, subgroup and sensitivity analyses were carried out. Funnel plots and Egger’s test assessed possible publication bias. Two-tailed P<0.05 suggested statistical significance. Every analysis was enabled by STATA 15.0 and Review Manager 5.4.

### 2.6 GRADE classification

Moreover, the evidence quality was rated utilizing the GRADE approach as “high,” “moderate,” “low,” or “very low” (24). Due to serious concerns regarding heterogeneity and imprecision, the quality of evidence for the relationships of NLR with OS, RFS, and pCR was rated as “very low.”

## 3 Results

### 3.1 Study characteristics

**Figure 1:**
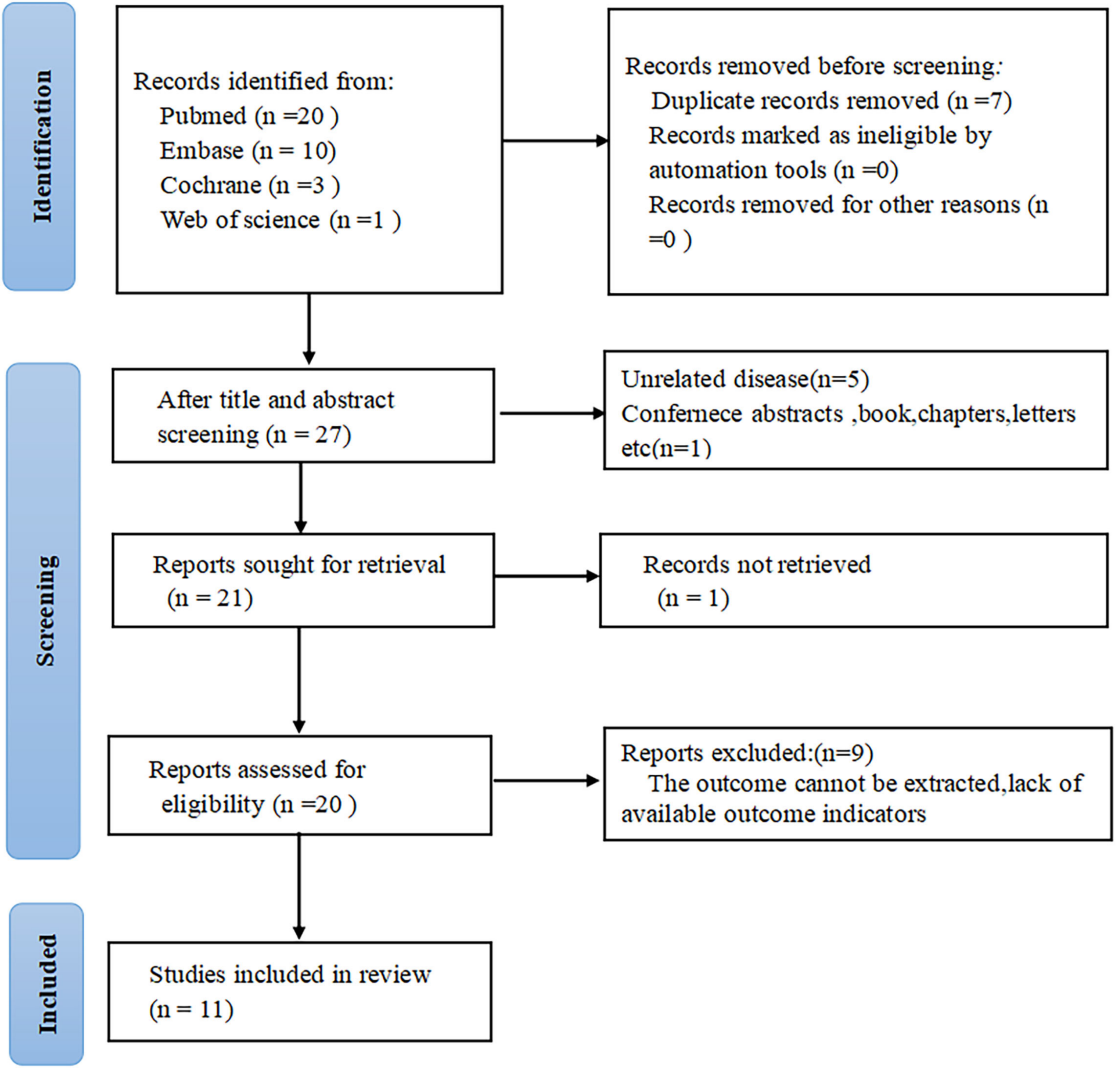
Flow chart of literature screening.

34 articles were initially identified through database searches. After removing seven duplicate publications, seven additional studies were excluded after title and abstract review. The full texts of the rest were checked, with nine excluded primarily due to partial or insufficient data relevant to the present analysis. Ultimately, eleven studies comprising 2,220 patients were encompassed (**Figure 1**).

Among the eleven eligible studies (25–33), six were conducted in Asian countries, while four originated from Western countries like the United Kingdom and the United States. Notably, all eleven studies were cohort studies, with nine being retrospective in design (5, 17, 27–33) and two being prospective (25, 26). All studies were published in English between 2016 and 2025. Each included study investigated EC sufferers who received neoadjuvant therapy, and participants were split into high and low NLR cohorts. Regarding NLR assessment, eight studies measured NLR before the initiation of neoadjuvant therapy, one study assessed preoperative NLR and two studies evaluated both pre-neoadjuvant and preoperative NLR levels. Based on these assessments, six studies examined the prognostic impact of NLR on OS, four studies focused on recurrence-free survival (RFS), and three studies investigated its association with pathologic complete response (pCR). The characteristics are presented in **Table 1**.

**Table 1:** Basic characteristics of the included literature.

Author	Year	Source of patients	Study type	Population	Age	Duration	Sample size	Treatments	TNM stage	NLR cut-off	Timing of detection
T. Grenader (25)	2016	UK and Australia	Prospective cohort	Esophageal gastric cancer	NA	2000-2005	908	NCT	NA	3	baseline
Hsueh, W. H (26)	2022	China	Prospective cohort	squamous cell carcinoma	56	2016-2017	123	NCRT	II-IV	3.1	baseline
Ji, W. H (27).	2016	China	Retrospective cohort	squamous cell carcinoma	56.6	2009-2012	41	NCT	NA	5	baseline
Li, C (17).	2021	China	Retrospective cohort	squamous cell carcinoma	54.77	2007-2016	127	NCRT	II-III	5.4	baseline
McLaren, P. J (29).	2017	US	Retrospective cohort	squamous cell carcinoma and adenocarcinoma of esophagus	NA	2005-2015	60	NCRT	NA	NA	baseline
Ohsawa, M (31).	2022	Japan	Retrospective cohort	squamous cell carcinoma	NA	2003-2018	163	NCRT	NA	4.5	baseline
Powell, Agmt (32)	2020	UK	Retrospective cohort	adenocarcinoma of esophagus	68	2010-2018	136	NCT	I-III	2.25	baseline
Tustumi, Francisco (33)	2020	NA	Retrospective cohort	squamous cell carcinoma and adenocarcinoma of esophagus	60.9	2009-2019	149	NCRT	NA	NA	baseline
Noble, F (30).	2013	UK	Retrospective cohort	Esophageal gastric cancer	67	2005-2010	246	NCT	II-III	2.5	baseline
Kim, J. Y (5).	2024	South Korea	Retrospective cohort	squamous cell carcinoma	63	2007-2017	123	NCRT	II-III	2.5	baseline
Kubo, K (28).	2024	Japan	Retrospective cohort	squamous cell carcinoma	64.1	2015-2020	144	NCT	I-IV	3	NA

NCRT, neoadjuvant chemoradiotherapy; NCT, neoadjuvant chemotherapy; NA, not available.

### 3.2 Study quality

11 eligible studies scored 7–9 on the NOS, indicating high methodological quality (**Supplementary Table 2**).Agreement between the two reviewers (LWM and JXH) for study selection was almost perfect (κ = 0.906, 95% CI 0.859 to 0.953, P < 0.001).

### 3.3 Meta-analysis results

#### 3.3.1 NLR and pCR

**Figure 2:**
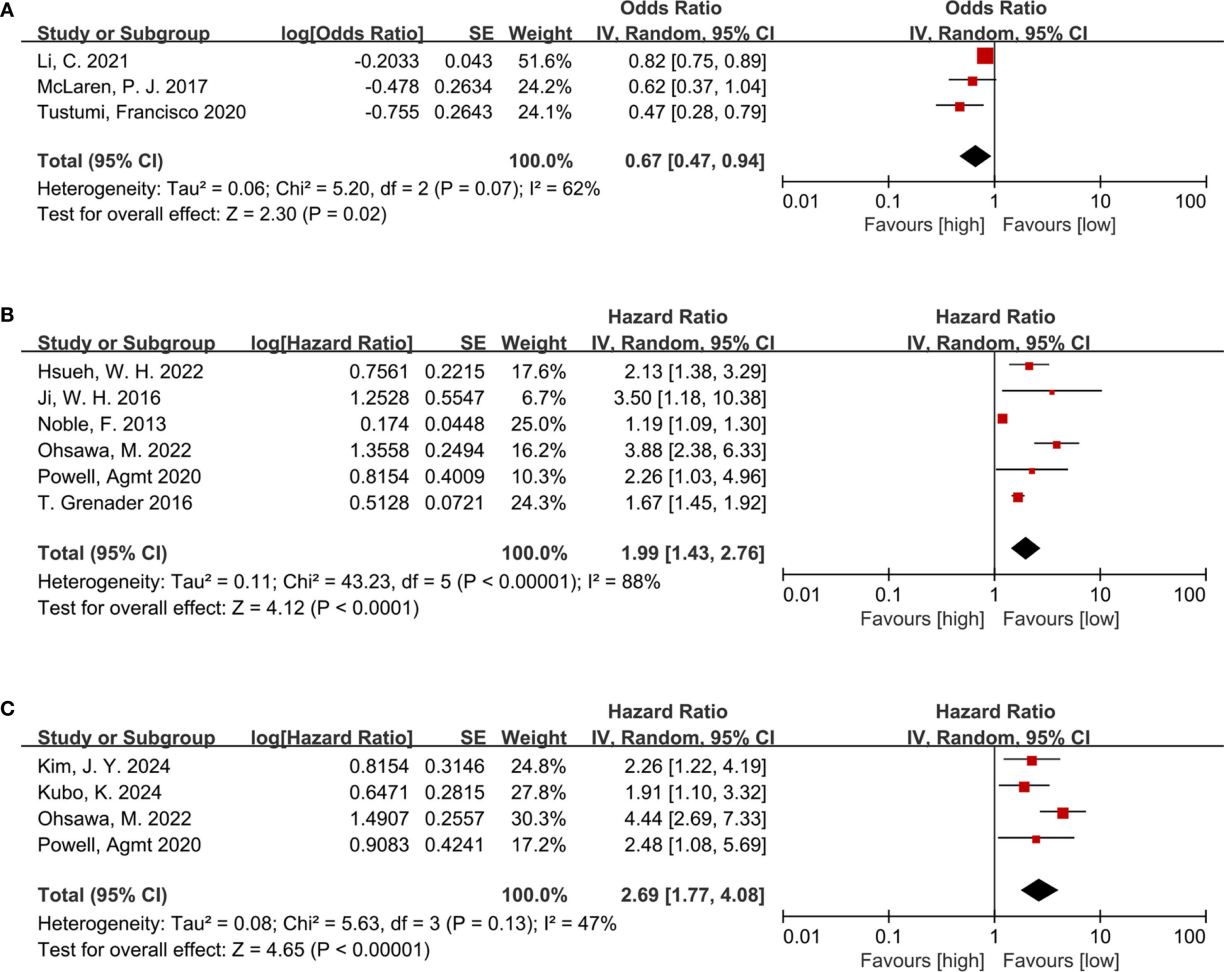
**(A)** Forest plots for the association between NLR and pCR; **(B)** Forest plots for the association between NLR and OS; **(C)** Forest plots for the association between NLR and RFS.

Three studies including 336 participants were analyzed to investigate the link of NLR to pathological complete response(pCR). Among EC patients receiving neoadjuvant therapy, a lower NLR was evidently linked to a risen pCR rate (OR = 0.67, 95% CI = 0.47–0.94, P = 0.02; I² = 62%) (**Figure 2A**), indicating an inverse relationship between NLR and treatment response.

#### 3.3.2 NLR and OS

The relationship between NLR and OS was examined across six cohort studies involving 1,617 participants. Due to substantial heterogeneity (I² = 88%, P < 0.00001), a random-effects model was leveraged (**Figure 2B**). A higher NLR was notably related to poorer OS among the EC population following neoadjuvant therapy (HR = 1.99, 95% CI = 1.43–2.76, P < 0.0001; I² = 88%) (**Figure 2B**).

#### 3.3.3 NLR and RFS

Four studies involving 566 participants provided data on the link of NLR to RFS. Consistent with the findings for OS, elevated NLR was markedly linked to shortened RFS (HR = 2.69, 95% CI = 1.77–4.08, P < 0.00001; I² = 47%) (**Figure 2C**) among patients treated with neoadjuvant therapy.

### 3.4 Subgroup analysis

To detect probable sources of heterogeneity, subgroup analyses regarding NLR were executed. Elevated NLR independently predicted poorer OS and RFS, regardless of age, follow-up duration, sample size, treatment modality and NLR cut-off value.

Firstly, subgroup analyses by sample size and treatment modality consistently showed a marked link of increased NLR to shortened OS (P < 0.05). Notably, the subgroup receiving NCRT showed a stronger association (HR = 2.84, 95% CI: 1.58–5.12, P = 0.0005) in contrast to the subgroup receiving NCT alone (HR = 1.58, 95% CI: 1.15–2.18, P = 0.004). Secondly, subgroup analysis by follow-up length indicated that among patients with follow-up less than 36 months, higher NLR was markedly linked to shorter OS (HR = 1.84, 95% CI: 1.43–2.35, P < 0.00001), while notable relation was not found in those with follow-up longer than 36 months (P = 0.09). Thirdly, age-based subgroup analysis showed that in patients younger than 60, elevated NLR evidently predicted less favorable OS (HR = 1.84, 95% CI: 1.43–2.35, P < 0.00001), whereas marked prognostic influence was not observed among those aged 60 or older (P = 0.09).Finally, based on the subgroup analysis of NLR cut-off values, both the subgroup with NLR cut-off value ≤ 3 and the subgroup with NLR cut-off value > 3 showed that higher NLR often indicated poor OS was statistically significant(P<0.05). Notably, the subgroup with NLR cut-off value>3 (HR = 2.91,95% CI = 1.87-4.52; P< 0.00001) showed a stronger correlation than the subgroup with NLR cut-off value ≤ 3 (HR = 1.49,95% CI = 1.09-2.03; P = 0.01).

Subgroup analyses for RFS based on the same parameters (sample size, follow-up time, age, treatment modality, and NLR cut-off value) all demonstrated a statistically significant relation of elevated NLR to shorter RFS (P < 0.05), indicating the robustness of NLR as a prognostic marker for RFS. Notably, a stronger link of risen NLR to reduced RFS was observed in patients with follow-up lasting over 36 months (HR = 3.09, 95% CI: 1.94–4.90, P < 0.00001) in contrast to those with shortened follow-up. Similarly, the NCRT subgroup showed a stronger correlation between elevated NLR and shortened RFS (HR = 3.25, 95% CI: 1.68–6.28, P = 0.0005) than the NCT subgroup. These findings are consistent with the OS results and warrant further investigation (**Table 2**).Otherwise, Subgroup analysis based on NLR cut-off values showed that there was a significant statistical difference between higher NLR and shorter RFS (P<0.05).NLR cut-off value > 3 subgroup had better predictive performance for RFS than NLR cut-off value ≤ 3 subgroup.

**Table 2:** Pooled HRs for OS and RFS in subgroup analyses.

Subgroup	OS				RFS			
	Study	HR [95%CI]	P value	I2	Study	HR [95%CI]	P value	I2
Total	6	1.99 [1.43, 2.76]	P<0.00001	88%	4	2.69 [1.77, 4.08]	P<0.00001	47%
Sample size								
≤130	2	2.28 [1.52, 3.41]	P<0.0001	0%	1	2.26 [1.22, 4.19]	P=0.01	NA
>130	4	1.86 [1.28, 2.70]	P=0.001	92%	3	2.82 [1.61,4.97]	P=0.0003	61%
Follow-up								
≤36mouths	3	1.84 [1.43, 2.35]	P<0.00001	27%	1	1.91 [1.10,3.32]	P=0.02	NA
>36mouths	3	2.13 [0.90, 5.04]	P=0.09	92%	3	3.09 [1.94, 4.90]	P<0.00001	47%
Age								
<60years old	3	1.84 [1.43, 2.35]	P<0.00001	27%	0	NA	NA	NA
>60years old	3	2.13 [0.90, 5.04]	P=0.09	92%	4	2.69 [1.77, 4.08]	P<0.00001	47%
Line of therapy								
NCRT	2	2.84 [1.58,5.12]	P=0.0005	69%	2	3.25 [1.68,6.28]	P=0.0005	64%
NCT	4	1.58 [1.15,2.18]	P=0.004	86%	2	2.07 [1.31,3.28]	P=0.002	0%
NLR cut-off								
NLR cut-off ≤ 3	3	1.49 [1.09, 2.03]	P=0.01	89%	3	2.14 [1.48,3.09]	P<0.0001	0%
NLR cut-off>3	3	2.91 [1.87, 4.52]	P<0.00001	41%	1	4.44 [3.69, 7.33]	P<0.00001	NA

### 3.5 Sensitivity analysis

**Figure 3:**
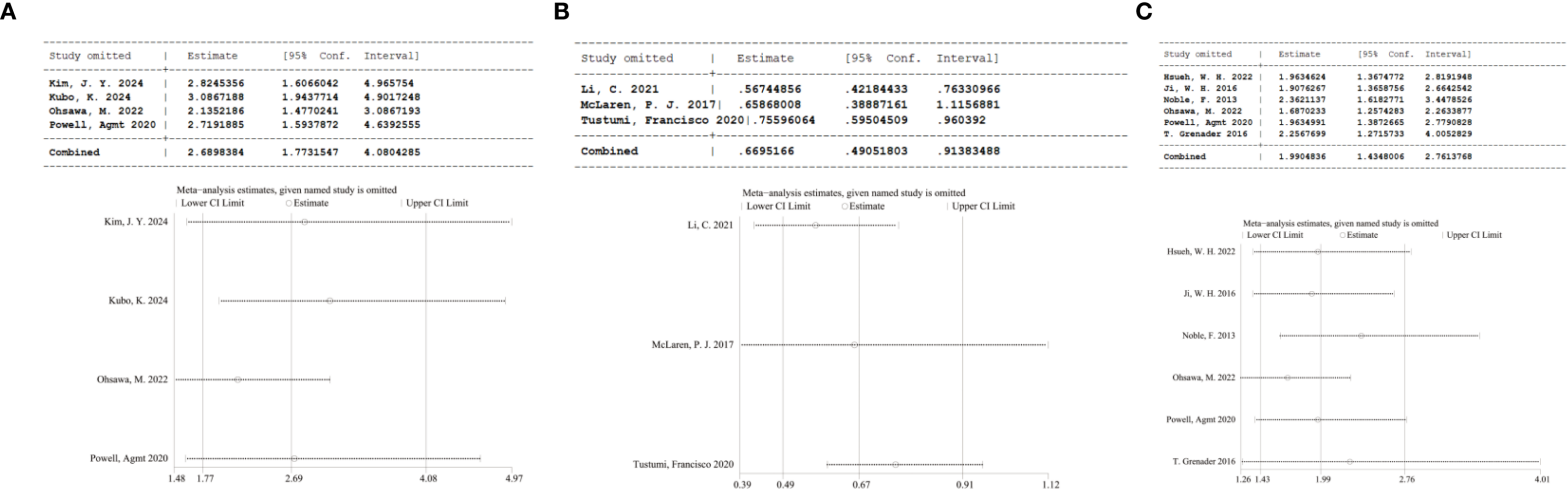
**(A)** Sensitivity analysis of RFS; **(B)** Sensitivity analysis of pCR; **(C)** Sensitivity analysis of OS.

Sensitivity analyses assessed the influence of separate studies on the overall effect size and evaluated the resulting robustness concerning NLR’s clinical relevance. For NLR and RFS (P = 0.13, I² = 47%), sequential exclusion of each study yielded consistent results within the original effect range, suggesting that no individual study disproportionately influenced our pooled estimate for RFS and confirming the reliability of the RFS findings (**Figure 3A**). For NLR and pCR, the exclusion of the study by McLaren P. J (2017) (29).altered the pooled result from statistically significant to insignificant (HR = 0.66, 95% CI: 0.39–1.12), indicating considerable effects of this study on the overall effect (**Figure 3B**). For NLR and OS (P < 0.00001, I² = 88%), sequential exclusion of individual studies also yielded consistent results within the original effect range, confirming that OS results were not disproportionately affected by any study and validating the reliability of the link of NLR to OS (**Figure 3C**).

### 3.6 Publication bias

**Figure 4:**
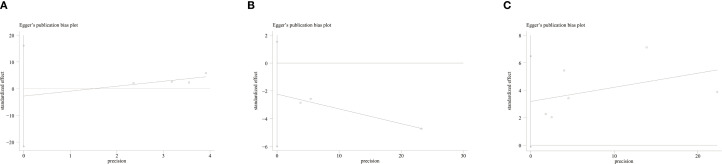
**(A)** Funnel plot for the evaluation of publication bias for RFS; **(B)** Funnel plot for the evaluation of publication bias for pCR; **(C)** Funnel plot for the evaluation of publication bias for OS.

Publication bias was detected via funnel plots and Egger’s test. The funnel plots for RFS (**Figure 4A**), pCR (**Figure 4B**), and OS (**Figure 4C**) did not show significant asymmetry, suggesting no obvious publication bias. Additionally, Egger’s test presented insignificant results regarding OS (P = 0.055), RFS (P = 0.591), and pCR (P = 0.084), further showing no publication bias.

### 3.7 GRADE classification

Through the GRADE approach, the evidence quality was classified as “high,” “moderate,” “low,” or “very low” as per standard criteria. This classification was used to assess the strength of recommendations for applying NLR as a prognostic indicator in EC sufferers receiving neoadjuvant therapy, thereby informing clinical decision-making (22). Due to substantial heterogeneity and imprecision, the quality of evidence for OS, RFS, and pCR was rated as very low (**Table 3**).

**Table 3:** GRADE rating of outcomes.

Outcomes	No. of studies	HR or OR	95%CI	*I* ^2^; P value	Risk of bias	Inconsistency	Indirectness	Imprecision P<0.001	Publication bias	Plausible confounding	Magnitude of effect	Dose-response gradient	GRADE
OS	6	1.99	1.43, 2.76	88%; P<0.0001	Serious risk	Serious inconsistency	No serious indirectness	No Serious imprecision	Undetected	Would not reduce effect	No	No	Very Low
RFS	4	2.69	1.77,4.08	47%;P<0.00001	Serious risk	No Serious inconsistency	No serious indirectness	No Serious imprecision	Undetected	Would not reduce effect	Yes	No	Very Low
pCR(OR)	3	0.67	0.47, 0.94	62%;P=0.02	Serious risk	serious inconsistency	No serious indirectness	Serious imprecision	Undetected	Would not reduce effect	No	No	Very Low

## 4 Discussion

Inflammation, a key feature of the tumor microenvironment, is crucial in cancer initiation, cell proliferation, angiogenesis, as well as metastasis during tumor progression (34, 35). Several studies have demonstrated that inflammatory markers hold prognostic value in cancer treatment and outcome prediction (36). Among them, NLR has been a proven prognostic indicator. In non-small cell lung, colorectal, gastric, ovarian cancers, hepatocellular and renal cell carcinomas (37–42), NLR is related to patient prognosis. Zhang CL et al. identified that NLR notably predicts survival and treatment response among the ovarian cancer population (43). Similarly, Mi HL et al.’s retrospective research on NLR in lung cancer proved that higher NLR can independently be adopted for forecasting reduced RFS (44). These findings suggest that NLR, a non-invasive, easily accessible, and cost-effective hematologic marker, may provide valuable prognostic information across various types of cancer.

This meta-analysis, which included 2,220 patients, demonstrated that NLR evidently correlates with OS, RFS, and pCR among the EC population. These findings have been validated by other studies. For instance, a 2021 study reported that among EC patients who received NCRT, those who achieved pCR exhibited evidently lower NLR levels (HR = 1.218, 95% CI = 1.050–1.414, P = 0.009) (45). Another meta-analysis published in 2024 unveiling NLR’s prognostic relevance in EC also proved that elevated NLR is markedly linked to worse OS (HR = 1.47, 95% CI = 1.29–1.67, P < 0.00001) (46). These consistent results reinforce our conclusion that NLR is a qualified and reliable prognostic indicator. As a cost-effective and easily obtainable hematological marker, NLR can facilitate the rapid construction of prognostic models and serve as a feasible component in the development of comprehensive prognostic prediction tools. Our subgroup analysis showed that NCRT offers greater prognostic benefits than NCT among the EC population. Furthermore, the subgroup analyses for RFS across various parameters demonstrated robust statistical power. Prognostic models constructed through this approach can assist clinicians in accurately assessing patient outcomes and adjusting treatment strategies in a timely manner, thereby potentially improving prognosis in high-risk individuals.

Our study elucidated the link of NLR to OS and RFS in EC sufferers and included subgroup analyses by sample size, follow-up duration, age, and treatment regimen. For OS, NLR showed a significant association among patients with a follow-up length of ≤36 months, while no marked link was observed in those with a follow-up lasting over 36 months. This may be due to increased loss to follow-up over longer durations, which can bias statistical analyses in retrospective studies and reduce the significance of subgroup findings with extended follow-up. Among patients aged <60, higher NLR was linked to shorter OS, whereas no significant association was found in patients aged >60. Rui et al. (47) demonstrated that the mortality risk of EC increases with age. Aging is accompanied by a gradual decline in immune function and cumulative exposure to risk factors, both of which contribute to higher mortality. This could account for the lack of statistical significance in NLR’s prediction value for OS among patients aged above 60. Notably, in the NCRT cohort, the prognostic effect of NLR (HR = 2.84, 95% CI = 1.58–5.12, P = 0.0005) was stronger than the NCT receivers(HR = 1.58, 95% CI = 1.15–2.18, P = 0.004). Similarly, for RFS, the link of NLR to prognosis was stronger in the NCRT group (HR = 3.25, 95% CI = 1.68–6.28, P = 0.0005) than in the NCT subgroup (HR = 2.07, 95% CI = 1.37–3.28, P = 0.002). Multiple studies have shown that perioperative NCRT, compared to NCT alone, offers superior benefits in terms of prognosis and postoperative complications among the EC population (48, 49). In our RFS subgroup analyses, all predefined subgroups demonstrated a significant link between NLR and patient outcomes. This consistent cross-subgroup finding further supports the reliability of NLR as a prognosis biomarker in EC, suggesting that its predictive value is largely independent of these clinical variables. Of note, our subgroup analysis also demonstrated that the subgroup with an NLR cut-off value > 3 exhibited better predictive performance for RFS compared to the subgroup with an NLR cut-off value ≤ 3. The superior predictive ability of an NLR cut-off > 3 may be attributed to the fact that a higher threshold more effectively identifies patients with severe systemic inflammation, which is closely associated with tumor recurrence.

This study explored the link of NLR to the prognosis of EC and conducted a comprehensive sensitivity analysis to ensure result robustness and reliability. All sensitivity analyses were performed utilizing a random-effects model. The stability of our pooled estimates was examined utilizing a leave-one-out approach. For NLR and RFS (I² = 47%, P = 0.13), heterogeneity was relatively low, and the effect sizes remained the same within the original range upon sequential exclusion of individual studies, indicating good stability in the RFS-related findings. In contrast, for NLR and pCR (I² = 62%, P = 0.07) and NLR and OS (I² = 88%, P < 0.00001), significant heterogeneity persisted even after stepwise study ostracization. This possibly arises from variations in study populations from various countries and regions, variability in study periods, and the relatively small sample sizes, which introduce both temporal and geographic heterogeneity. Taken together, the sensitivity analysis suggests that, despite differences in patient characteristics and study design, the prognostic value of NLR remains robust. Probable publication bias was examined utilizing Egger’s test. No publication bias existed in the analyses of OS, RFS, and pCR in relation to NLR. Furthermore, GRADE assessments were executed and evidence was rated as “very low” for each outcome. This was mainly due to the observational nature of eligible studies, which are inherently prone to methodological limitations. Therefore, Additional high-quality prospective research is necessitated for verifying and strengthening the evidence base for the prognostic utility of NLR in this setting. Ultimately, the goal is to provide clinicians with better tools for making informed decisions on the post-neoadjuvant treatment of EC sufferers. From the perspective of outcome stability, our findings further confirm that elevated NLR is significantly associated with worse OS and RFS, while its association with lower pCR rates is less stable due to the influence of individual studies; this is consistent with the sensitivity analysis result that excluding the McLaren P. J (2017). study altered the statistical significance of the pCR-related association.

Neutrophils exhibit an intricate role in the tumor microenvironment. Once recruited by tumor cells into the tumor milieu, neutrophils become tumor-associated neutrophils (TANs): N1 and N2 phenotypes. TANs support tumor growth primarily by releasing proliferative factors, suppressing T-cell activity, and promoting tumor angiogenesis (50). In contrast, lymphocytes are central to antitumor immunity. CD8^+^ T cells are cytotoxic against tumor cells via the secretion of IFN-γ and TNF-α, while CD4^+^ T cells activate antigen-presenting cells and enhance the cytotoxic effects of both CD8^+^ T cells and NK cells, thereby inhibiting tumor growth and metastasis (51, 52). Lymphocyte count decrease is often connected with poor prognosis among the EC population (53). A risen NLR usually reflects a relative elevation in neutrophils and/or a drop in lymphocytes. Neutrophils suppress the activity of lymphocytes and NK cells, diminishing antitumor immune responses, while also contributing to systemic inflammation through the release of pro-inflammatory mediators. Conversely, lymphopenia weakens the ability of the immune system to target and eliminate tumor cells, thereby facilitating tumor invasion and metastasis (54). Lymphocytes play crucial roles in restraining tumor cell proliferation, and metastasis, and promoting cytotoxicity and apoptosis, whereas neutrophils facilitate tumor progression by producing growth factors, chemokines, and proteases (55). When elevated neutrophil counts and reduced lymphocyte counts co-occur, the predictive value for tumor progression may be further enhanced. Thus, NLR is regarded as a potential prognostic biomarker for EC sufferers following neoadjuvant therapy. Despite presenting valuable insights, this meta-analysis has limitations. Most incorporated studies were retrospective, with only two being prospective. The retrospective design may introduce uncontrolled confounding factors that could bias the conclusions. Moreover, most were conducted in Asia and Europe, suggesting potential geographic limitations. Therefore, broad generalizations to non-studied populations should be approached with caution. Further research is required to validate NLR’s prognostic relevance among the EC population undergoing neoadjuvant therapy in other geographic settings. Additionally, variability in the cutoff values and timing of NLR measurement may introduce heterogeneity and confounding effects that are difficult to control. Differences in treatment regimens across studies may also contribute to the inconsistency in results. Therefore, subsequent researchers should focus on standardizing when NLR is measured, selecting consistent treatment approaches, and determining clear, measurable NLR thresholds.

## 5 Conclusion

In conclusion, following neoadjuvant therapy, elevated NLR is markedly linked to poor prognosis among the EC population, including reduced OS and RFS and lower pCR. Therefore, NLR is a low-cost, non-invasive, and easily accessible biomarker for forecasting the prognosis of EC sufferers. It can aid clinicians in timely developing optimal treatment plans based on the potential risk of poor outcomes. Notably, this hematological biomarker may be valuable for forecasting the prognosis of EC in underdeveloped or resource-limited regions, especially in clinical settings where genomic monitoring is not feasible. However, due to some inevitable limitations and confounding factors in the included studies, it is recommended to carry out a multi-center prospective study to standardize the NLR measurement time, such as baseline and cutoff values before treatment, in order to confirm the conclusions of this study.

## Data Availability

The original contributions presented in the study are included in the article/**Supplementary Material**. Further inquiries can be directed to the corresponding author.

## Supplementary material

Supplementary Table 1 Search strategy.

## References

[1] Yang X Tang Z Li J Jiang J . Esophagus cancer and essential trace elements. Front Public Health. (2022) 10:1038153. doi: 10.3389/fpubh.2022.1038153, PMID: 36466456 PMC9709130

[2] Bray F Laversanne M Sung H Ferlay J Siegel RL Soerjomataram I . Global cancer statistics 2022: GLOBOCAN estimates of incidence and mortality worldwide for 36 cancers in 185 countries. CA Cancer J Clin. (2024) 74:229–63. doi: 10.3322/caac.21834, PMID: 38572751

[3] Sung H Ferlay J Siegel RL Laversanne M Soerjomataram I Jemal A . Global cancer statistics 2020: GLOBOCAN estimates of incidence and mortality worldwide for 36 cancers in 185 countries. CA Cancer J Clin. (2021) 71:209–49. doi: 10.3322/caac.21660, PMID: 33538338

[4] Arnold M Abnet CC Neale RE Vignat J Giovannucci EL McGlynn KA . Global burden of 5 major types of gastrointestinal cancer. Gastroenterology. (2020) 159:335–49.e15. doi: 10.1053/j.gastro.2020.02.068, PMID: 32247694 PMC8630546

[5] Kim JY Yun JK Kim YH Park SI Lee JH Jung HY . Prognostic impact of inflammation-based factors in patients with esophageal squamous cell carcinoma achieving pathological complete response after neoadjuvant chemoradiotherapy followed by surgery. Ann Surg Oncol. (2024) 31:6662–72. doi: 10.1245/s10434-024-15678-y, PMID: 38954089

[6] Yuan Y Chen LQ . Interpretation of update on the AJCC esophageal cancer staging system, eighth edition. Zhonghua Waike Zazhi. (2017) 55:109–13. doi: 10.3760/cma.j.issn.0529-5815.2017.02.007, PMID: 28162209

[7] Shi X Zhao H Yu J Cai P Zhou S Yang N . Changes in PD-1 expression on T lymphocyte subsets and related immune indicators before and after definitive chemoradiotherapy for esophageal squamous cell carcinoma. Ann Med. (2025) 57:2445190. doi: 10.1080/07853890.2024.2445190, PMID: 39713872 PMC11703528

[8] Kato K Machida R Ito Y Daiko H Ozawa S Ogata T . Doublet chemotherapy, triplet chemotherapy, or doublet chemotherapy combined with radiotherapy as neoadjuvant treatment for locally advanced oesophageal cancer (JCOG1109 NExT): a randomised, controlled, open-label, phase 3 trial. Lancet. (2024) 404:55–66. doi: 10.1016/S0140-6736(24)00745-1, PMID: 38876133

[9] Li J Ma S . History and current situation of neoadjuvant treatment for locally advanced esophageal cancer. Thorac Cancer. (2021) 12:2293–9. doi: 10.1111/1759-7714.14069, PMID: 34254738 PMC8410532

[10] Tang H Wang H Fang Y Zhu JY Yin J Shen YX . Neoadjuvant chemoradiotherapy versus neoadjuvant chemotherapy followed by minimally invasive esophagectomy for locally advanced esophageal squamous cell carcinoma: a prospective multicenter randomized clinical trial. Ann Oncol. (2023) 34:163–72. doi: 10.1016/j.annonc.2022.10.508, PMID: 36400384

[11] Zhang G Zhang C Sun N Xue L Yang Z Fang L . Neoadjuvant chemoradiotherapy versus neoadjuvant chemotherapy for the treatment of esophageal squamous cell carcinoma: a propensity score-matched study from the National Cancer Center in China. J Cancer Res Clin Oncol. (2022) 148:943–54. doi: 10.1007/s00432-021-03659-7, PMID: 34013382 PMC11801005

[12] Lewis S Lukovic J . Neoadjuvant therapy in esophageal cancer. Thorac Surg Clin. (2022) 32:447–56. doi: 10.1016/j.thorsurg.2022.06.003, PMID: 36266032

[13] Chen DS Mellman I . Elements of cancer immunity and the cancer-immune set point. Nature. (2017) 541:321–30. doi: 10.1038/nature21349, PMID: 28102259

[14] Zhi X Jiang K Shen Y Su X Wang K Ma Y . Peripheral blood cell count ratios are predictive biomarkers of clinical response and prognosis for non-surgical esophageal squamous cell carcinoma patients treated with radiotherapy. J Clin Lab Anal. (2020) 34:e23468. doi: 10.1002/jcla.23468, PMID: 32681567 PMC7595892

[15] Mosca M Nigro MC Pagani R De Giglio A Di Federico A . Neutrophil-to-lymphocyte ratio (NLR) in NSCLC, gastrointestinal, and other solid tumors: immunotherapy and beyond. Biomolecules. (2023) 13:1803. doi: 10.3390/biom13121803, PMID: 38136673 PMC10741961

[16] Mouchli M Reddy S Gerrard M Boardman L Rubio M . Usefulness of neutrophil-to-lymphocyte ratio (NLR) as a prognostic predictor after treatment of hepatocellular carcinoma.” Review article. Ann Hepatol. (2021) 22:100249. doi: 10.1016/j.aohep.2020.08.067, PMID: 32896610

[17] Li C Lin JW Yeh HL Chuang CY Chen CC . Good prediction of treatment responses to neoadjuvant chemoradiotherapy for esophageal cancer based on preoperative inflammatory status and tumor glucose metabolism. Sci Rep. (2021) 11:11626. doi: 10.1038/s41598-021-90753-y, PMID: 34078965 PMC8172631

[18] Anand S Bhati G Gurram R Gnanasekaran S Kate V Pottakkat B . Does neutrophil-to-lymphocyte ratio (NLR) predict pathologic response to neoadjuvant chemoradiotherapy in patients with esophageal squamous cell carcinoma? J gastrointestinal Cancer. (2021) 52:659–65. doi: 10.1007/s12029-020-00445-5, PMID: 32607960

[19] Page MJ McKenzie JE Bossuyt PM Boutron I Hoffmann TC Mulrow CD . The PRISMA 2020 statement: an updated guideline for reporting systematic reviews. BMJ. (2021) 372:n71. doi: 10.1136/bmj.n71, PMID: 33782057 PMC8005924

[20] Wells GS O’Connell D Peterson J Welch V Losos M Tugwell P . The Newcastle-Ottawa Scale (NOS) for assessing the quality of nonrandomised studies in meta-analyses. Ottawa: Ottawa Hospital Research Institute (2014).

[21] Higgins JP Altman DG Gøtzsche PC Jüni P Moher D Oxman AD . The Cochrane Collaboration’s tool for assessing risk of bias in randomised trials. BMJ. (2011) 343:d5928. doi: 10.1136/bmj.d5928, PMID: 22008217 PMC3196245

[22] Landis JR Koch GG . The measurement of observer agreement for categorical data. Biometrics. (1977) 33:159–74. doi: 10.2307/2529310 843571

[23] Higgins JP Thompson SG Deeks JJ Altman DG . Measuring inconsistency in meta-analyses. BMJ. (2003) 327:557–60. doi: 10.1136/bmj.327.7414.557, PMID: 12958120 PMC192859

[24] Guyatt G Oxman AD Akl EA Kunz R Vist G Brozek J . GRADE guidelines: 1. Introduction-GRADE evidence profiles and summary of findings tables. J Clin Epidemiol. (2011) 64:383–94. doi: 10.1016/j.jclinepi.2010.04.026, PMID: 21195583

[25] Grenader T Waddell T Peckitt C Oates J Starling N Cunningham D . Prognostic value of neutrophil-to-lymphocyte ratio in advanced oesophago-gastric cancer: exploratory analysis of the REAL-2 trial. Ann Oncol. (2016) 27:687–92. doi: 10.1093/annonc/mdw012, PMID: 26787231

[26] Hsueh WH Hsueh SW Yeh KY Hung YS Ho MM Lin SY . Albumin and neutrophil-to-lymphocyte ratio score in neoadjuvant concurrent chemoradiotherapy for esophageal cancer: comparison with prognostic nutritional index. In Vivo (Athens Greece). (2022) 36:2400–8. doi: 10.21873/invivo.12973, PMID: 36099141 PMC9463917

[27] Ji WH Jiang YH Ji YL Li B Mao WM . Prechemotherapy neutrophil : lymphocyte ratio is superior to the platelet : lymphocyte ratio as a prognostic indicator for locally advanced esophageal squamous cell cancer treated with neoadjuvant chemotherapy. Dis esophagus. (2016) 29:403–11. doi: 10.1111/dote.12322, PMID: 25625421

[28] Kubo K Igaue S Utsunomiya D Kubo Y Kanematsu K Kurita D . Preoperative neutrophil-to-lymphocyte ratio predicts recurrence of esophageal squamous cell carcinoma after neoadjuvant triplet chemotherapy. Gen Thorac Cardiovasc surgery. (2024) 72:802–9. doi: 10.1007/s11748-024-02053-7, PMID: 38913280

[29] McLaren PJ Bronson NW Hart KD Vaccaro GM Gatter KM Thomas CR Jr. . Neutrophil-to-lymphocyte and platelet-to-lymphocyte ratios can predict treatment response to neoadjuvant therapy in esophageal cancer. J gastrointestinal Surg. (2017) 21:607–13. doi: 10.1007/s11605-016-3351-4, PMID: 28083838

[30] Noble F Hopkins J Curtis N Kelly JJ Bailey IS Byrne JP . The role of systemic inflammatory and nutritional blood-borne markers in predicting response to neoadjuvant chemotherapy and survival in oesophagogastric cancer. Med Oncol (Northwood London England). (2013) 30:596. doi: 10.1007/s12032-013-0596-6, PMID: 23690267

[31] Ohsawa M Hamai Y Emi M Ibuki Y Kurokawa T Yoshikawa T . Neutrophil-to-lymphocyte ratio as a predictor of postoperative recurrence and prognosis in oesophageal squamous cell carcinoma. Anticancer Res. (2022) 42:1499–507. doi: 10.21873/anticanres.15622, PMID: 35220245

[32] Powell A Chin C Coxon AH Chalishazar A Christian A Roberts SA . Neutrophil to lymphocyte ratio as a predictor of response to neoadjuvant chemotherapy and survival in oesophageal adenocarcinoma. BJS Open. (2020) 4:416–23. doi: 10.1002/bjs5.50277, PMID: 32232963 PMC7260416

[33] Tustumi F Takeda FR Viyuela MS da Cruz Junior JB Brandão A Sallum RAA . The value of cellular components of blood in the setting of trimodal therapy for esophageal cancer. J Surg Oncol. (2020) 121:784–94. doi: 10.1002/jso.25778, PMID: 31762058

[34] Zhang J Veeramachaneni N . Targeting interleukin-1β and inflammation in lung cancer. biomark Res. (2022) 10:5. doi: 10.1186/s40364-021-00341-5, PMID: 35086565 PMC8796434

[35] Yang D Sun X Wang H Wistuba II Wang H Maitra A . TREM2 depletion in pancreatic cancer elicits pathogenic inflammation and accelerates tumor progression via enriching IL-1b(+) macrophages. Gastroenterology. (2025) 168(6):1153–69. doi: 10.1053/j.gastro.2025.01.244, PMID: 39956331 PMC12103993

[36] Yurou C . Prognostic analysis of primary gastrointestinal diffuse large B-cell lymphoma based on systemic inflammatory response index. CNKI: Shandong University (2023).

[37] Huang D Ren Q Xie L Chen Y Li C Su X . Association between airway microbiota and systemic inflammation markers in non-small cell lung cancer patients. Sci Rep. (2025) 15:3539. doi: 10.1038/s41598-025-86231-4, PMID: 39875410 PMC11775180

[38] Pacholczak-Madej R Drobniak A Grela-Wojewoda A Calik J Viegas NV Tusień-Małecka D . Prognostic significance of peripheral blood biomarkers in patients with advanced renal cell carcinoma treated with nivolumab and ipilimumab-a polish multicenter, observational study. Clin Exp Med. (2025) 25:45. doi: 10.1007/s10238-024-01544-4, PMID: 39849293 PMC11759459

[39] Xu N Zhang JX Zhang JJ Huang Z Mao LC Zhang ZY . The prognostic value of the neutrophil-to-lymphocyte ratio (NLR) and platelet-to-lymphocyte ratio (PLR) in colorectal cancer and colorectal anastomotic leakage patients: a retrospective study. BMC Surg. (2025) 25:57. doi: 10.1186/s12893-024-02708-5, PMID: 39910526 PMC11796187

[40] Yu C Jiang H Wang L Jiang Z Jin C . Baseline (derived) neutrophil-lymphocyte ratio associated with survival in gastroesophageal junction or gastric cancer treated with ICIs. Front Oncol. (2025) 15:1404695. doi: 10.3389/fonc.2025.1404695, PMID: 39926278 PMC11802431

[41] Li SX Bao Y Wang TC . Subcutaneous adipose tissue/neutrophil-to-lymphocyte ratio is a potential biomarker in patients with hepatocellular carcinoma undergoing liver resection. Sci Prog. (2024) 107:368504241304195. doi: 10.1177/00368504241304195, PMID: 39668576 PMC11639030

[42] Dinca AL Diaconu A Birla RD Coculescu BI Dinca VG Tudorache IS . Systemic inflammatory markers - prognostic value in ovarian cancer. Acta Endocrinol (Buchar). (2024) 20:162–9. doi: 10.4183/aeb.2024.162, PMID: 39845755 PMC11750215

[43] Zhang CL Jiang XC Li Y Pan X Gao MQ Chen Y . Independent predictive value of blood inflammatory composite markers in ovarian cancer: recent clinical evidence and perspective focusing on NLR and PLR. J Ovarian Res. (2023) 16:36. doi: 10.1186/s13048-023-01116-2, PMID: 36759864 PMC9912515

[44] Mi HL Wei WL Zhang DH Liang HY Yue CF Xu JN . Neutrophil-to-lymphocyte ratio, platelet-to-lymphocyte ratio, and prognostic nutritional index as prognostic markers for lung carcinoma. Br J Hosp Med (Lond). (2024) 85:1–13. doi: 10.12968/hmed.2024.0270, PMID: 39475029

[45] Wu Y Chen J Zhao L Li Q Zhu J Yang H . Prediction of pathologic response to neoadjuvant chemoradiotherapy in patients with esophageal squamous cell carcinoma incorporating hematological biomarkers. Cancer Res Treat. (2021) 53:172–83. doi: 10.4143/crt.2020.594, PMID: 32898941 PMC7812014

[46] Wu X Liu S Li F Chen Y . Association between preoperative neutrophil-to-lymphocyte ratio and the survival outcomes of esophageal cancer patients underwent esophagectomy: a systematic review and meta-analysis. Front Oncol. (2024) 14:1404711. doi: 10.3389/fonc.2024.1404711, PMID: 39224809 PMC11366628

[47] Rui X Wei CC Li J Zhang C-L Jiang X-C Li Y . Age-period-cohort model analysis on mortality trend of esophageal cancer in China from 2007 to 2021. Chin J Cancer Prev Treat. (2025) 32:70–7. doi: 10.1186/s13048-023-01116-2, PMID: 36759864 PMC9912515

[48] Pabon CM Spieler B Li JJ Ajani J Hosein PJ Blum Murphy M . Is it time to retire preoperative radiation for localized esophageal and gastro-esophageal adenocarcinoma? Oncologist. (2025) 30:1–13. doi: 10.12968/hmed.2024.0270, PMID: 39846982 PMC11756299

[49] Dong J Li C Wang B Li Y Wang S Cui H . Prognostic analysis of esophageal cancer patients after neoadjuvant therapy. Front Immunol. (2025) 16:1553086. doi: 10.3389/fimmu.2025.1553086, PMID: 40061941 PMC11885245

[50] Lu T Li W . Neutrophil engulfment in cancer: friend or foe? Cancers (Basel). (2025) 17:1404711. doi: 10.3389/fonc.2024.1404711, PMID: 39941753 PMC11816126

[51] St Paul M Ohashi PS . The roles of CD8(+) T cell subsets in antitumor immunity. Trends Cell Biol. (2020) 30:695–704. doi: 10.16073/j.cnki.cjcpt.2025.02.02, PMID: 32624246

[52] Liu Q Wang L Lin H Wang Z Wu J Guo J . Tumor-specific CD4(+) T cells restrain established metastatic melanoma by developing into cytotoxic CD4(-) T cells. Front Immunol. (2022) 13:875718. doi: 10.3389/fimmu.2022.875718, PMID: 35784297 PMC9243303

[53] Wang X Gao Y Wang J Chen L Zhang X Chen M . Predictive role of elevated neutrophil-lymphocyte ratio for bone metastasis in esophageal cancer. Technol Cancer Res Treat. (2024) 23:15330338241272043. doi: 10.1177/15330338241272043, PMID: 39149934 PMC11329970

[54] Platini H Ferdinand E Kohar K Prayogo SA Amirah S Komariah M . Neutrophil-to-lymphocyte ratio and platelet-to-lymphocyte ratio as prognostic markers for advanced non-small-cell lung cancer treated with immunotherapy: A systematic review and meta-analysis. Med (Kaunas). (2022) 58:384. doi: 10.3390/medicina58081069, PMID: 36013536 PMC9413376

[55] Kemuriyama K An J Motoyama S Nagaki Y Yamaguchi T Sato Y . Squamous cell carcinoma-derived G-CSF promotes tumor growth and metastasis in mice through neutrophil recruitment and tumor cell proliferation, associated with poor prognosis of the patients. Genes Cells. (2023) 28:573–84. doi: 10.1111/gtc.13051, PMID: 37248626

