# Supplementary material for: Prognostic value of neutrophil to lymphocyte ratio in patients with esophagus cancer receiving neoadjuvant therapy: a systematic review and meta-analysis
Source: Front Immunol. 2025 Oct 6;16:1615962. doi: 10.3389/fimmu.2025.1615962 (PMC12535995; doi:10.3389/fimmu.2025.1615962)

The search formula is as follows

PUBMED-20，Search：

**(((((("Esophageal Neoplasms"[Mesh]) AND (((((((((Esophageal Neoplasm) OR (Esophagus Neoplasm)) OR (Esophagus Neoplasms)) OR (Cancer of Esophagus)) OR (Esophageal Cancer)) OR (Esophageal Cancers)) OR (Cancer of the Esophagus)) OR (Esophagus Cancer)) OR (Esophagus Cancers))) AND ("Neutrophils"[Mesh])) AND ("Lymphocytes"[Mesh])) AND ("Neoadjuvant Therapy"[Mesh])) AND (((((((((((((((((((((((((((Neoadjuvant Therapies) OR (Neoadjuvant Treatment)) OR (Neoadjuvant Treatments)) OR (Neoadjuvant Chemotherapy)) OR (Neoadjuvant Chemotherapies)) OR (Neoadjuvant Chemotherapy Treatment)) OR (Neoadjuvant Chemotherapy Treatments)) OR (Neoadjuvant Chemoradiotherapy)) OR (Neoadjuvant Chemoradiotherapies)) OR (Neoadjuvant Chemoradiation)) OR (Neoadjuvant Chemoradiations)) OR (Neoadjuvant Chemoradiation Therapy)) OR (Neoadjuvant Chemoradiation Therapies)) OR (Neoadjuvant Chemoradiation Treatment)) OR (Neoadjuvant Chemoradiation Treatments)) OR (Neoadjuvant Systemic Therapy)) OR (Neoadjuvant Systemic Therapies)) OR (Neoadjuvant Systemic Treatment)) OR (Neoadjuvant Systemic Treatments)) OR (Neoadjuvant Radiotherapy)) OR (Neoadjuvant Radiotherapies)) OR (Neoadjuvant Radiation)) OR (Neoadjuvant Radiations)) OR (Neoadjuvant Radiation Treatment)) OR (Neoadjuvant Radiation Treatments)) OR (Neoadjuvant Radiation Therapy)) OR (Neoadjuvant Radiation Therapies))) AND (ratio)**

**
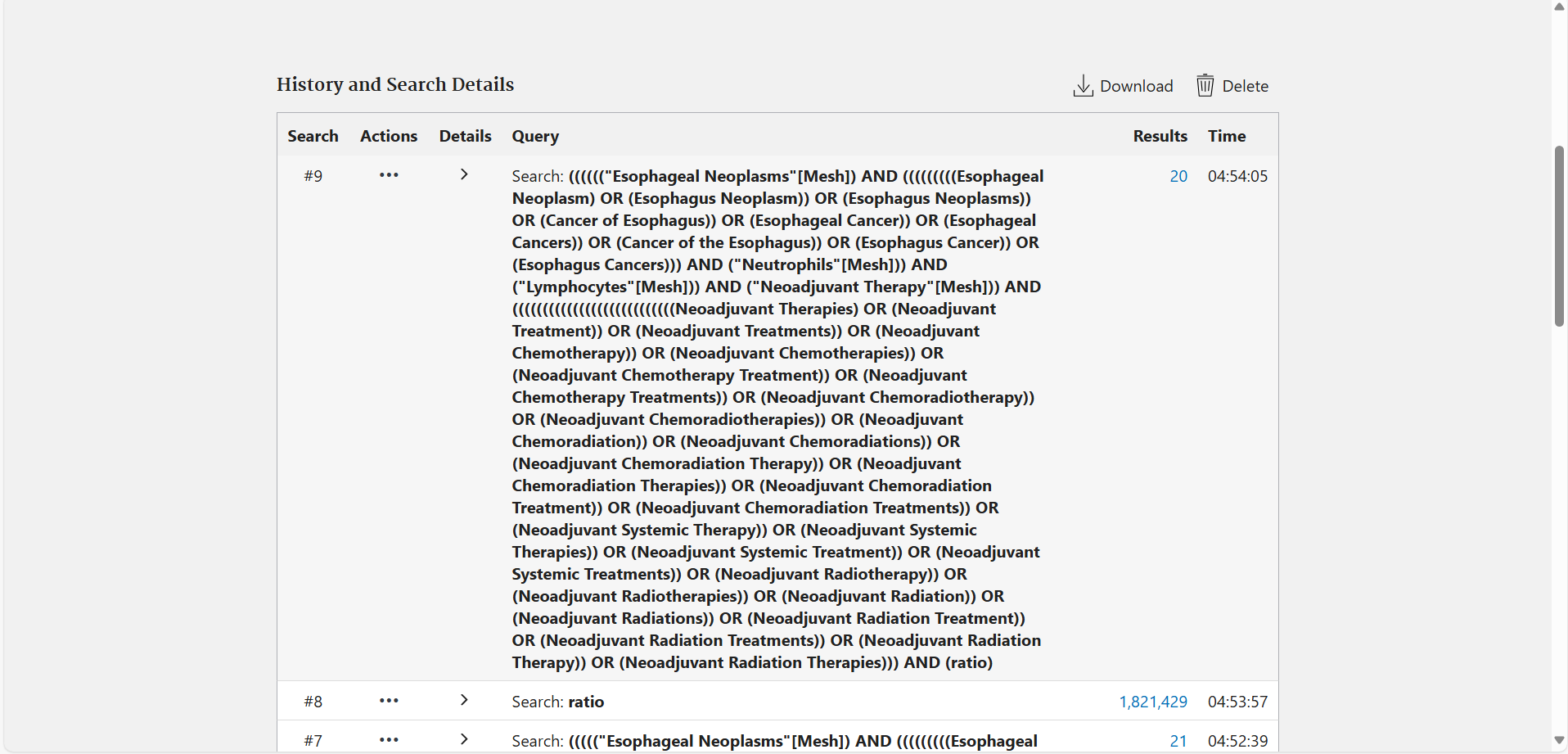
**

Embase-10

**(((((("Esophageal Neoplasms") AND (((((((((Esophageal Neoplasm) OR (Esophagus Neoplasm)) OR (Esophagus Neoplasms)) OR (Cancer of Esophagus)) OR (Esophageal Cancer)) OR (Esophageal Cancers)) OR (Cancer of the Esophagus)) OR (Esophagus Cancer)) OR (Esophagus Cancers))) AND ("Neutrophils")) AND ("Lymphocytes")) AND ("Neoadjuvant Therapy")) AND (((((((((((((((((((((((((((Neoadjuvant Therapies) OR (Neoadjuvant Treatment)) OR (Neoadjuvant Treatments)) OR (Neoadjuvant Chemotherapy)) OR (Neoadjuvant Chemotherapies)) OR (Neoadjuvant Chemotherapy Treatment)) OR (Neoadjuvant Chemotherapy Treatments)) OR (Neoadjuvant Chemoradiotherapy)) OR (Neoadjuvant Chemoradiotherapies)) OR (Neoadjuvant Chemoradiation)) OR (Neoadjuvant Chemoradiations)) OR (Neoadjuvant Chemoradiation Therapy)) OR (Neoadjuvant Chemoradiation Therapies)) OR (Neoadjuvant Chemoradiation Treatment)) OR (Neoadjuvant Chemoradiation Treatments)) OR (Neoadjuvant Systemic Therapy)) OR (Neoadjuvant Systemic Therapies)) OR (Neoadjuvant Systemic Treatment)) OR (Neoadjuvant Systemic Treatments)) OR (Neoadjuvant Radiotherapy)) OR (Neoadjuvant Radiotherapies)) OR (Neoadjuvant Radiation)) OR (Neoadjuvant Radiations)) OR (Neoadjuvant Radiation Treatment)) OR (Neoadjuvant Radiation Treatments)) OR (Neoadjuvant Radiation Therapy)) OR (Neoadjuvant Radiation Therapies))) AND (ratio)**

**
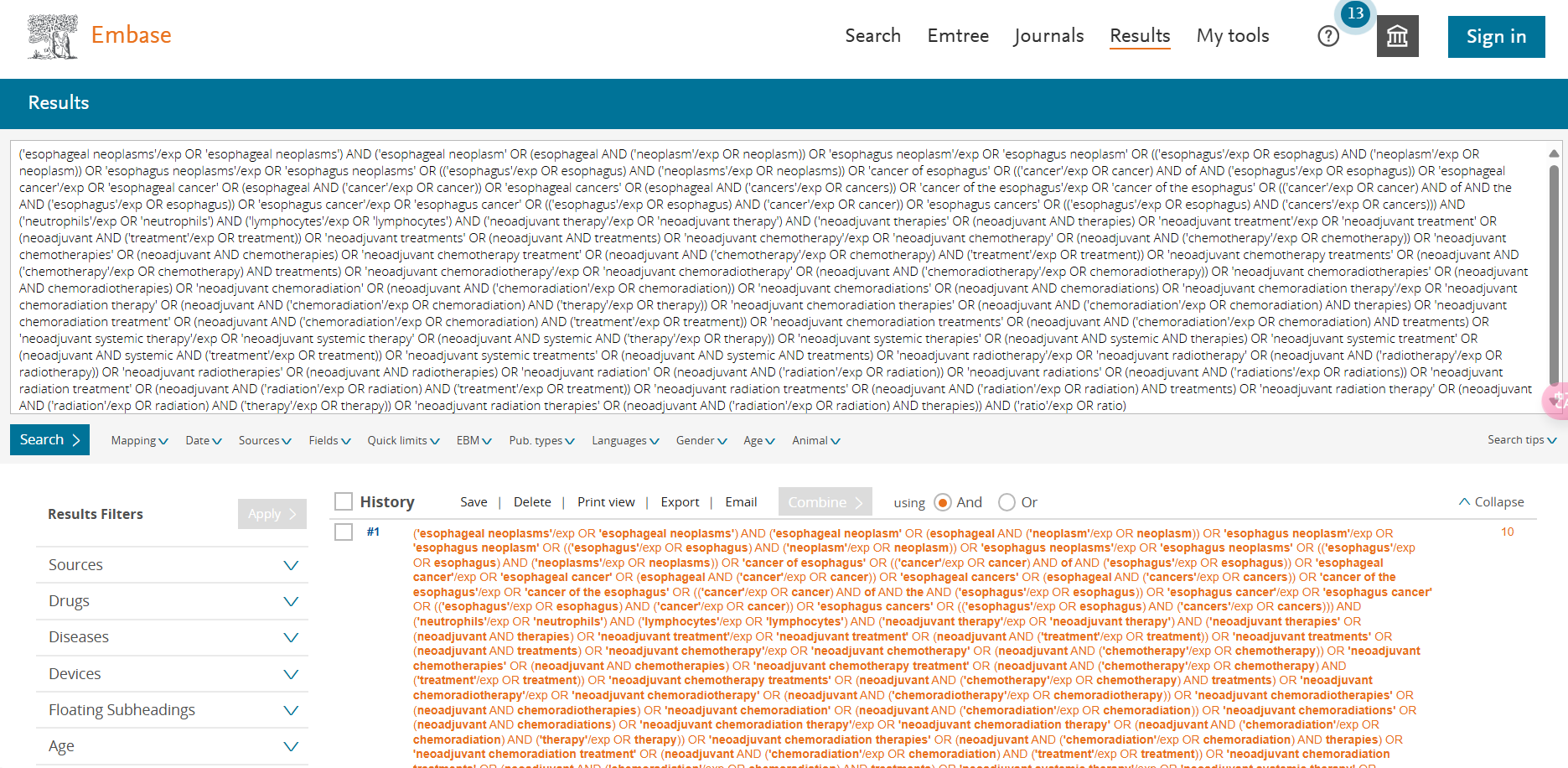
**

**Web of science-1**

**((((((Esophageal Neoplasms) AND (((((((((Esophageal Neoplasm) OR (Esophagus Neoplasm)) OR (Esophagus Neoplasms)) OR (Cancer of Esophagus)) OR (Esophageal Cancer)) OR (Esophageal Cancers)) OR (Cancer of the Esophagus)) OR (Esophagus Cancer)) OR (Esophagus Cancers))) AND (Neutrophils)) AND (Lymphocytes])) AND (Neoadjuvant Therapy)) AND (((((((((((((((((((((((((((Neoadjuvant Therapies) OR (Neoadjuvant Treatment)) OR (Neoadjuvant Treatments)) OR (Neoadjuvant Chemotherapy)) OR (Neoadjuvant Chemotherapies)) OR (Neoadjuvant Chemotherapy Treatment)) OR (Neoadjuvant Chemotherapy Treatments)) OR (Neoadjuvant Chemoradiotherapy)) OR (Neoadjuvant Chemoradiotherapies)) OR (Neoadjuvant Chemoradiation)) OR (Neoadjuvant Chemoradiations)) OR (Neoadjuvant Chemoradiation Therapy)) OR (Neoadjuvant Chemoradiation Therapies)) OR (Neoadjuvant Chemoradiation Treatment)) OR (Neoadjuvant Chemoradiation Treatments)) OR (Neoadjuvant Systemic Therapy)) OR (Neoadjuvant Systemic Therapies)) OR (Neoadjuvant Systemic Treatment)) OR (Neoadjuvant Systemic Treatments)) OR (Neoadjuvant Radiotherapy)) OR (Neoadjuvant Radiotherapies)) OR (Neoadjuvant Radiation)) OR (Neoadjuvant Radiations)) OR (Neoadjuvant Radiation Treatment)) OR (Neoadjuvant Radiation Treatments)) OR (Neoadjuvant Radiation Therapy)) OR (Neoadjuvant Radiation Therapies))) AND (ratio)**

Cochrane-3

**(((((Esophageal Neoplasms) AND (((((((((Esophageal Neoplasm) OR (Esophagus Neoplasm)) OR (Esophagus Neoplasms)) OR (Cancer of Esophagus)) OR (Esophageal Cancer)) OR (Esophageal Cancers)) OR (Cancer of the Esophagus)) OR (Esophagus Cancer)) OR (Esophagus Cancers))) AND (Neutrophils)) AND (Lymphocytes)) AND (Neoadjuvant Therapy)) AND (((((((((((((((((((((((((((Neoadjuvant Therapies) OR (Neoadjuvant Treatment)) OR (Neoadjuvant Treatments)) OR (Neoadjuvant Chemotherapy)) OR (Neoadjuvant Chemotherapies)) OR (Neoadjuvant Chemotherapy Treatment)) OR (Neoadjuvant Chemotherapy Treatments)) OR (Neoadjuvant Chemoradiotherapy)) OR (Neoadjuvant Chemoradiotherapies)) OR (Neoadjuvant Chemoradiation)) OR (Neoadjuvant Chemoradiations)) OR (Neoadjuvant Chemoradiation Therapy)) OR (Neoadjuvant Chemoradiation Therapies)) OR (Neoadjuvant Chemoradiation Treatment)) OR (Neoadjuvant Chemoradiation Treatments)) OR (Neoadjuvant Systemic Therapy)) OR (Neoadjuvant Systemic Therapies)) OR (Neoadjuvant Systemic Treatment)) OR (Neoadjuvant Systemic Treatments)) OR (Neoadjuvant Radiotherapy)) OR (Neoadjuvant Radiotherapies)) OR (Neoadjuvant Radiation)) OR (Neoadjuvant Radiations)) OR (Neoadjuvant Radiation Treatment)) OR (Neoadjuvant Radiation Treatments)) OR (Neoadjuvant Radiation Therapy)) OR (Neoadjuvant Radiation Therapies))) AND (ratio)**


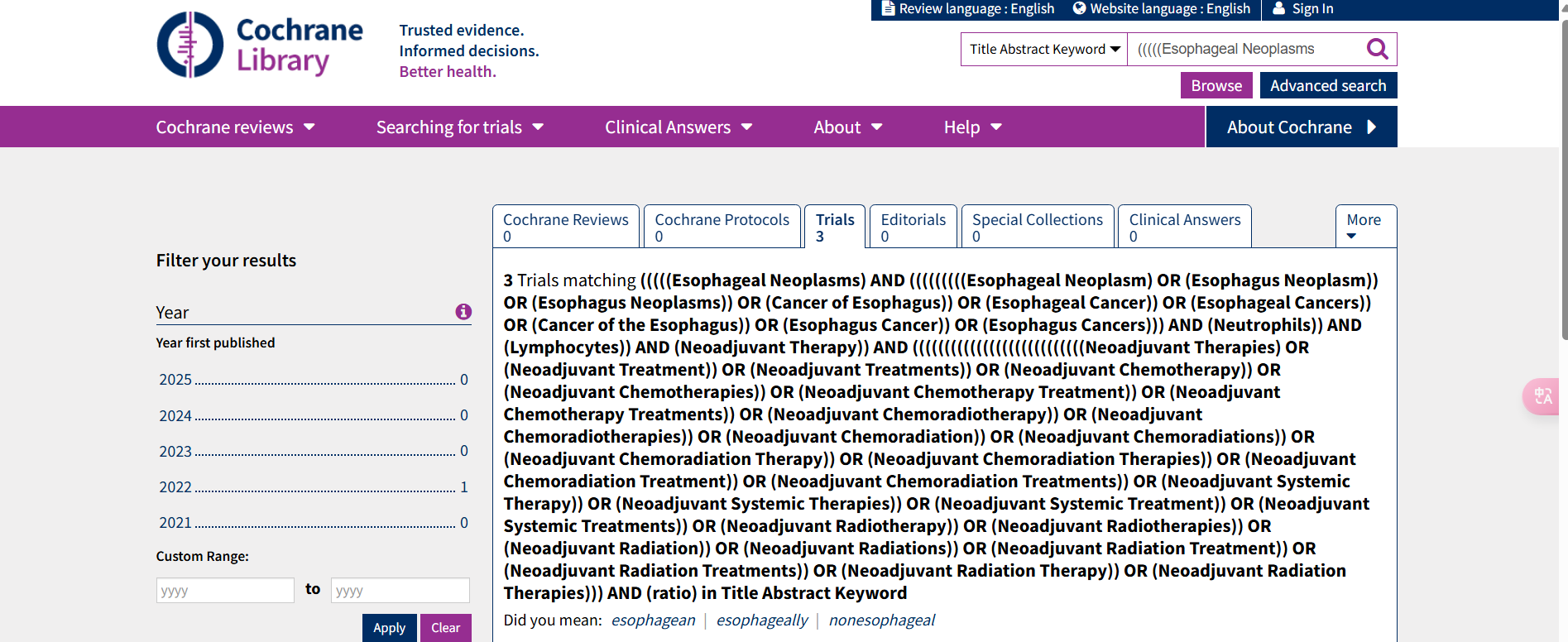

Supplement: Supplementary Table 1 — Search strategy. [file Table1.docx]
