# Supplementary material for: Prognostic value of neutrophil to lymphocyte ratio in patients with esophagus cancer receiving neoadjuvant therapy: a systematic review and meta-analysis
Source: Front Immunol. 2025 Oct 6;16:1615962. doi: 10.3389/fimmu.2025.1615962 (PMC12535995; doi:10.3389/fimmu.2025.1615962)
Supplement: Supplementary file 2 [file Table2.docx]

| Supplementary Table 2. Quality evaluation of the eligible studies with Newcastle–Ottawa scale. | | | | | | | | | |
| --- | --- | --- | --- | --- | --- | --- | --- | --- | --- |
| Study | Selection | | | | Comparability | | Outcome | | |
|  | Representative*ness | Selection of  non*exposed | Ascertainment  of exposure | Outcome not present at start | Comparability on most important factors | Comparability on other risk factors | Assessment of outcome | Long enough follow*up (median≥1 year) | Adequacy  (completeness) of follow*up |
| T. Grenader2016 | * | * | * | * | - | - | * | * | * |
| Hsueh, W. H.2022 | * | * | * | * | * | * | * | * | * |
| Ji, W. H.2016 | * | * | * | * | * | * | * | * | * |
| Li, C.2021 | * | * | * | * | * | - | * | * | * |
| McLaren, P. J.2017 | * | * | * | * | * | - | * | - | * |
| Ohsawa, M.2022 | * | * | * | * | * | - | * | * | * |
| Powell, Agmt2020 | * | * | * | * | * | - | * | * | * |
| Tustumi, Francisco2020 | * | * | * | * | * | - | * | * | * |
| Noble, F.2013 | * | * | * | * | * | - | * | * | * |
| Kim, J. Y.2024 | * | * | * | * | * | - | * | * | * |
| Kubo, K.2024 | * | * | * | * | * | - | * | * | * |
|  |  |  |  |  |  |  |  |  |  |
|  |  |  |  |  |  |  |  |  |  |
